# Ferroptosis in Myocardial Fibrosis: Mechanisms and Therapeutic Insights

**DOI:** 10.3390/antiox15010070

**Published:** 2026-01-06

**Authors:** Xuefeng Lin, Weijun Li, Jiahao Ye, Lin Li

**Affiliations:** College of Chinese Medicine, Hunan University of Chinese Medicine, Changsha 410208, China; linxf21@stu.hnucm.edu.cn (X.L.); 20243622@stu.hnucm.edu.cn (W.L.)

**Keywords:** ferroptosis, myocardial fibrosis, lipid peroxidation, glutathione metabolism, Nrf2 signaling pathway

## Abstract

Myocardial fibrosis (MF) is a common pathological feature of diverse cardiac disorders and is a key driving factor of cardiac dysfunction. It is marked by excessive deposition of extracellular matrix (ECM) proteins, particularly collagen type I and III, and a prolonged activation of cardiac fibroblasts. However, the molecular drivers of this process remain undetermined. Ferroptosis is an iron-catalyzed, lipid-peroxidation-dependent mode of regulated cell death. Research indicates that ferroptosis is significantly involved in the onset and advancement of MF; consequently, developing therapies that selectively modulate ferroptosis presents a promising direction of treatment options. Therefore, this paper systematically discusses the mechanisms associated with ferroptosis to explore the link between ferroptosis and MF from multiple dimensions, including iron metabolism disorders, lipid peroxidation, imbalance of glutathione metabolism, and the dysregulated activation of ferroptosis regulatory pathways, to provide innovative perspectives for the study of the specific molecular mechanisms and treatment of MF. **Method:** By retrieving the literature on the mechanism of ferroptosis in MF published in PubMed and Web of Science databases from 2020 to July 2025, the mechanism of action was systematically analyzed and reviewed.

## 1. Introduction

Myocardial fibrosis (MF) is a pathological remodeling process that results from myocardial injury. It is characterized by reduced cardiac function, stiffening of the heart muscles, and even heart failure [1,2]. During this condition, excessive collagen accumulates and is deposited in the heart tissue. This phenomenon is commonly observed in late-stage heart conditions like atherosclerosis, coronary heart disease, and myocardial infarction [3]. The prolonged proliferation and aberrant activation of cardiac fibroblasts (CFs) are the core drivers of the mechanism. Activated CFs and their differentiated myofibroblasts serve as the primary effector cells in myocardial fibrosis (MF) and are the principal producers of matrix proteins. These proteins contribute to significant deposition and disorganization of the extracellular matrix (ECM), which in turn causes aberrant remodeling of the collagenous network. This process ultimately leads to dilatation of the interstitial matrix of the myocardium and structural fibrotic alterations [4,5]. These pathologic changes not only disrupt the mechanical properties of the heart but also severely impair cardiac function. Epidemiological analysis of fibrotic diseases has found that they cause 800,000 deaths per year worldwide, with a majority resulting from MF [6]. Despite extensive investigations into the significance of MF in the progression of heart disease, the specific molecular mechanisms underlying the pathophysiology remain an active area of investigation.

Ferroptosis is a recently discovered type of iron-dependent programmed cell death. This type of cell death is marked by the uncontrolled peroxidation of phospholipid-based polyunsaturated fatty acids (PL-PUFA) within cell membranes, ultimately leading to the disruption of cell membrane structure and the disintegration of the cell [7]. According to recent research, ferroptosis and fibrotic disease are strongly linked, and the aberrant activation of ferroptosis may aggravate cardiomyocyte injury, thus facilitating the progression of fibrosis [8,9]. A study observing MF phenotype, Fe^2+^ deposition, and lipid oxidation products (LOPs) accumulation in a mouse model of heart failure identified that these three factors were key pathological features [10]. Additionally, several investigations revealed the crucial involvement of ferroptosis-related genes and signaling pathways (for example, nuclear receptor coactivator 4 (NCOA4), cystine/glutamate antiporter system Xc^-^ (system Xc^-^)/glutathione (GSH)/glutathione peroxidase 4 (GPX)) in ferroptosis, with the system Xc^-^/GSH/GPX4 pathway playing a particularly important role in MF [11,12]. Therefore, this article discusses and investigates various facets of the link between ferroptosis and MF, including iron metabolism disorders, lipid peroxidation, imbalance of the GSH metabolism, and the dysregulated activation of ferroptosis regulatory pathways. The objective is to offer novel perspectives that establish the specific molecular mechanisms and treatment of MF.

## 2. Occurrence and Mechanisms of Ferroptosis

**Figure 1:**
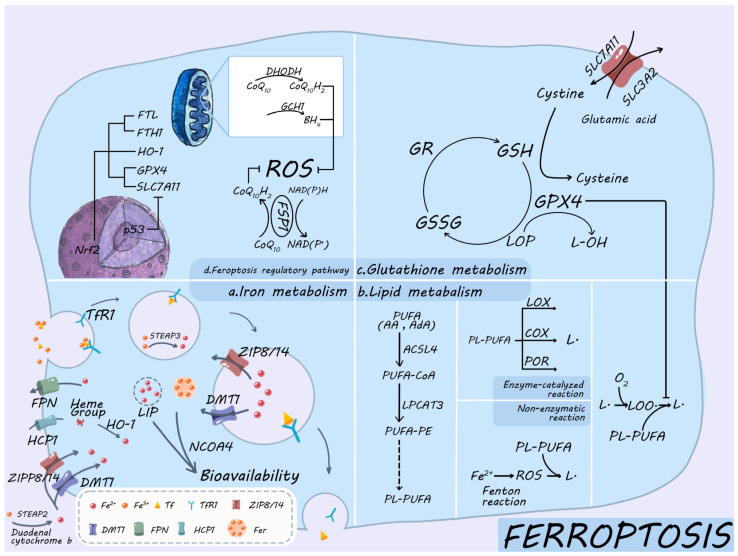
(**a**) depicts the iron metabolism pathway, encompassing iron absorption, utilization, storage, and excretion. The absorption pathways include the transferrin (Tf)-dependent pathway, the Tf-independent pathway, and the heme-iron uptake pathway. (**b**) depicts the lipid peroxidation pathway in lipid metabolism, encompassing the initiation, propagation, and termination phases of lipid peroxidation. The propagation phase is categorized into enzymatic and non-enzymatic forms. (**c**) shows the glutathione metabolic pathway. (**d**) depicts the regulatory pathways of ferroptosis, including the p53-Nrf2 signaling network, the FSP1/CoQ_10_ pathway, the DHODH/CoQ_10_ pathway, and the GCH1/BH_4_ pathway. Abbreviations: Tf: Transferrin; TfR1: Transferrin Receptor 1; ZIP8/14: Zinc-iron transport protein 8/14, solute carrier family 39 member a8/14; DMT1: Divalent metal transporter 1; FPN: Iron transport protein, Ferroportin; HCP1: Heme carrier protein 1; Fer: Ferritin; HO-1: Enzyme heme oxygenase-1; LIP: Labile iron pool; NCOA4: Nuclear receptor coactivator 4; STEAP2: Six-transmembrane epithelial antigen of prostate 2; STEAP3: Six-transmembrane epithelial antigen of prostate 3; PUFA: Polyunsaturated Fatty Acid; AA: Arachidonic acid; AdA: Adrenic acid; ACSL4: Coa synthetase long-chain family member 4; LPCAT3: Lysophosphatidylcholine acyltransferase 3; PE: Phosphatidylethanolamines; CoA: Coenzyme A; PL-PUFA: Phospholipid-based polyunsaturated fatty acids; LOX: Lipoxygenase; COX: Cyclooxygenase; POR: Cytochrome P450 oxidoreductase; ROS: Oxygen species; SLC7A11:Solute carrier family 7 member 11; SLC3A2: Solute carrier family 3 member 2; GSH: Glutathione; GSSG: Glutathione disulfide; GR: Glutathione reductase; LOP: Lipid oxidation products; Nrf2: Nuclear factor erythroid 2–related factor 2; p53: Tumor protein 53; FTL: Ferritin light polypeptide; FTH1: Ferritin heavy chain 1; GPX4: Glutathione peroxidase 4; DHODH: Dihydroorotate dehydrogenase; CoQ_10_: Coenzyme Q10, Ubiquinone; CoQ_10_H_2_: Reduced Coenzyme Q10, Ubiquinol; GCH1: GTP cyclohydrolase 1; BH_4_: tetrahydrobiopterin; FSP1:Ferroptosis suppressor protein 1.

Ferroptosis is an iron-dependent, lipid peroxidation-driven programmed cell death, and its core mechanism can be summarized as a triple reaction of iron overload-lipid peroxidation (LPO)-antioxidant system imbalance [13]. Numerous metabolic pathways, such as the ferroptosis regulatory pathway, lipid metabolism, GSH metabolism, and iron metabolism, work synergistically to modulate this mechanism (Figure 1).

### 2.1. Iron Metabolism

Maintaining cellular iron homeostasis is a tightly controlled process that balances iron absorption, utilization, storage, and excretion through iron metabolism [14]. Iron uptake is mediated mainly via three routes, namely, the Transferrin (Tf)-dependent pathway, the Tf-independent pathway, and the heme-iron uptake pathway [15]. When iron enters the cells as ferrous iron (Fe^2+^), it is either stored as ferritin (Fer) or becomes part of the labile iron pool (LIP). To release iron from Fer, the cell degrades Fer through ferritinophagy, which is mediated by cargo receptor NCOA4 [16]. The available Fe^2+^ from this process can participate in subsequent bioavailability or is effluxed by the membrane iron transport protein (Ferroportin, FPN) [17,18]. The iron-responsive element/Iron-regulatory protein (IRE/IRP) system senses and regulates iron levels in this process, thereby maintaining intracellular iron homeostasis [19].

#### 2.1.1. Iron Intake

Tf serves as a key mediator for the uptake of iron into the cells through cytosolization in response to the transferrin receptor (TfR). This is the primary pathway for iron uptake in most cells [20]. The Tf-dependent iron uptake pathway, also termed TfR-mediated holo-Tf endocytosis process, is composed of seven steps, namely, binding, endocytosis, acidification, dissociation, reduction, translocation, and recycling [21]. Holo-Tf (iron-saturated transferrin) binds to the Transferrin Receptor 1 (TfR1) located on the cell surface at physiological pH ≈ 7.4, forming a complex. Subsequently, this complex undergoes internalization through lattice protein-mediated endocytosis, initiating a process that facilitates the delivery of iron into the cell [22]. Due to the acidic environment inside the nuclear endosome (pH ≈ 5.5), Fe^3+^ is released from its complex, a process facilitated by H^+^-ATPase activity; subsequently, STEAP3, a six-transmembrane epithelial antigen of the prostate 3 located in the endosome, reduces Fe^3+^ to Fe^2+^ [23]. STEAP is a family of transmembrane proteins that includes STEAP1-4. STEAP3 is crucial for iron reduction within endosomes, while STEAP2 primarily participates in iron reduction at the cell membrane within the Tf-independent iron uptake pathway. Additionally, STEAP2 works in synergy with STEAP3 to regulate iron metabolism [24]. Zinc-iron transport protein 8/14 (solute carrier family 39 member a8/14, ZIP8/14) or divalent metal transporter 1 (DMT1) then carries the Fe^2+^ from the endosomal membrane to the cytoplasm. DMT1 contains four isoforms, including the isoform DMT1A-I, which is distributed on the cell membrane and participates in the Tf-independent iron uptake pathway [23]. Thereafter, the generated dissociated apo-Tf in endosomes then binds strongly to TfR1 and is returned to the cell surface via cytokinesis [25]. Apo-Tf is then dissociated from TfR1 and reenters the extracellular environment to continue participating in cyclic iron transport. Metal transporter proteins on the cell membrane are primarily responsible for the Tf-independent iron uptake pathway. Duodenal cytochrome b and STEAP2 convert Fe^3+^ to Fe^2+^, which is then translocated into the cell via DMT1 and ZIP8/14 on the cell membrane. The activity of this pathway becomes significantly enhanced during an iron deficiency state [26,27,28]. In the heme-iron uptake pathway, heme iron is taken up by the cell as a porphyrin ring-bound molecule, unlike non-heme iron, which must first be reduced from its ferric Fe^3+^ to its ferrous Fe^2+^ state to be absorbed by the cell [29]. Heme iron enters small intestinal cells in its native porphyrin ring-bound form via the heme carrier protein 1 (HCP1). Once inside the cell, the heme is metabolized by the enzyme heme oxygenase-1 (HO-1) [29].

#### 2.1.2. Iron Storage, Release, and Utilization

Iron storage and release are mainly mediated by Fer, a process that depends on the interplay between its heavy-chain subunit (FTH1) and its light-chain partner (FTL) [30]. FTH1 exhibits ferroxidase activity, facilitating the conversion of Fe^2+^ to Fe^3+^, and subsequently stores it within the internal cavity. In contrast, FTL offers acidic residues to aid iron nucleation. Part of the free Fe^2+^ other than Fer storage is coordinated by GSH to form cytoplasmic LIP and binds to polycytidylic acid-binding proteins (which function as iron chaperones) to participate in subsequent bioavailability [31]. NCOA4 fundamentally releases stored iron for utilization. This receptor is a cytoplasmic autophagy receptor that interacts with Fer to maintain iron homeostasis in cells [19]. NCOA4 binds to the FTH1 subunit of Fer to trigger ferritinophagy, leading to the release of Fer-stored Fe^3+^, reducing Fe^2+^ in response to STEAP3 (this autophagic process is enhanced under iron-deficient conditions and promotes the release of intracellular Fe^2+^ and its supply to the mitochondria, thereby sustaining mitochondrial function) [32,33]. Iron efflux is then facilitated by FPN, thereby maintaining intracellular iron homeostasis [18].

#### 2.1.3. Regulation of Iron Metabolism

The IRE/IRP system tightly controls intracellular iron metabolism. In an iron-deficient environment, IRP-1 and IRP-2 are activated to reduce iron storage and maintain intracellular iron homeostasis by regulating mRNA translation and promoting TfR1 expression to increase iron uptake. This activation also suppresses Fer expression to decrease iron storage and maintain intracellular iron homeostasis [19]. Additionally, the ferredoxin/FPN pathway controls iron homeostasis at the systemic level [25].

### 2.2. Lipid Peroxidation

The process of lipid peroxidation (LPO) involves the formation of lipid reactive oxygen species (L-ROS) and LOP. It occurs in cellular membranes rich in PL-PUFA and is driven by reactive oxygen species (ROS) [34]. The process consists of two phases: initiation and propagation.

#### 2.2.1. Initiation of Lipid Peroxidation

The LPO initiation phase centers on the process of PL-PUFA generation, transport, and eventual binding to membranes. During ferroptosis, membrane peroxidation initially accumulates in endoplasmic reticulum membranes and later in plasma membranes [35]. Ferroptosis is triggered by the peroxidation of two essential fatty acyl groups, arachidonic acid (AA) and adrenic acid (AdA), at an endoplasmic reticulum -based oxygenation center. Specifically, only phosphatidylethanolamines (PEs) containing these fatty acids are targeted for oxidation [36]. In the presence of acyl-CoA synthetase long-chain family member 4 (ACSL4), AA and AdA are bound to coenzyme A (CoA) to form AA-CoA and AdA-CoA [37]. Subsequent esterification to phosphatidylethanolamine by the action of lysophosphatidylcholine acyltransferase 3 (LPCAT3) generates arachidonic acid (PE-AA) and adrenic acid (PE-AdA) [38]. PE-AA and PE-AdA are then integrated into individual biological membranes through a series of lipid transport and assembly. PL-PUFA (especially PE-AA and PE-AdA) are primary targets of LPO in cell membranes and participate in the subsequent chain reaction [39].

#### 2.2.2. The Occurrence of Lipid Peroxidation

LPO occurs in three distinct stages, namely, initiation, propagation, and termination [34]. The initiation phase is characterized by the production of lipid radicals (L·), a process that involves both non-enzymatic and enzymatic pathways. In the non-enzymatic pathway, disruptions in iron metabolism result in the production of hydroxyl radicals (-OH) via the Fenton reaction, which occurs in the presence of excess Fe^2+^. This reaction interacts with PL-PUFA, leading to the creation of numerous lipid-free radicals (L·) and facilitating the breakdown of generated LOPs. This further intensifies the buildup of free radicals and the chain reaction [40]. The enzymatic pathway is mediated by lipoxygenase (LOX), cyclooxygenase (COX), and cytochrome P450 oxidoreductase (POR), which collectively participate in the oxidative metabolism of various lipids [41,42]. The oxidation of PL-PUFA at the 15-carbon site is directly catalyzed by ALOX15, a crucial member of the LOX family, producing numerous lipid radicals (L·) and their metabolite 15-HETE, which substantially accelerates the onset of LPO [43]. In addition, the ALOX15 pathway interacts and synergizes with COX and POR to amplify inflammation, drive LPO, and modulate the cellular redox state. This synergy creates a feedback loop that sustains and intensifies the inflammatory process, placing ALOX15 in a central position for initiating LPO [44]. Once in the propagation phase, lipid free radicals (L·) combine with oxygen to generate lipid peroxyl radicals (LOO·). This (LOO·) then removes a hydrogen atom from a neighboring PUFA, creating a new lipid free radical(L·) and lipid hydroperoxides (LOOHs), which perpetuates the chain reaction [34]. In the presence of Fe^2+^, LOOH is further decomposed into products such as alkoxyl radicals, significantly accelerating the reaction propagation [34]. The termination phase relies on the role of the intracellular antioxidant system, which is represented by antioxidants such as GPX4 and vitamin E that effectively block the continuation of the chain reaction and restore the redox balance of the cell membrane by trapping and reducing lipid peroxyl radicals (LOO·) [45,46]. In conclusion, LPO is a chain reaction initiated and sustained by free radicals and modulated by multiple enzymatic systems. The initiation and propagation phases promote each other, and the termination phase relies on antioxidant mechanisms for inhibition and is thus important in pathological processes such as ferroptosis.

### 2.3. Glutathione Metabolism

GSH metabolism is primarily governed by system Xc^-^ and GPX4, which together form the GSH/GPX4/GSSG metabolic cycle and the system Xc^-^/GSH/GPX4 pathway. GSH is the major non-enzymatic antioxidant in the cell, and its homeostatic maintenance is dependent on system Xc^-^ mediated uptake of cystine and subsequent redox cycling [47,48]. System Xc^-^ consists of the catalytic light-chain Solute carrier family 7 member 11 (SLC7A11) and the stabilizing heavy-chain Solute carrier family 3 member 2 (SLC3A2); the former swaps one cytosolic glutamate for one extracellular cystine, whereas the latter secures the transporter’s membrane stability and activity [49,50]. Inside the cytosol, cystine undergoes rapid reduction to cysteine, thereby supplying the rate-limiting precursor for GSH biosynthesis. While neutralizing ROS and LOP, GSH is itself converted to GSH disulfide; glutathione reductase (GR) promptly regenerates the reduced form, sustaining the intracellular GSH pool [51,52]. This process maintains a high intracellular GSH/GSSG ratio, which is an important indicator of redox status [53,54]. GPX4, as a selenocysteine-dependent antioxidant enzyme, is required to reduce LOP to nontoxic lipocalciferol with the synergistic effect of GSH, while generating GSSG, thus effectively blocking the LPO chain reaction [55]. Thus, the system Xc^-^/GSH/GPX4 pathway constitutes a frontline antioxidant shield that safeguards lipid bilayers against ferroptotic damage.

### 2.4. Regulatory Pathways of Ferroptosis

In addition to controlling the expression of system Xc^-^ (primarily SLC7A11) and GPX4, the regulatory pathway of ferroptosis maintains cellular antioxidant capacity independently of the system Xc^-^/GSH/GPX4 pathway, which collectively constitutes the cellular antioxidant system. The representative pathways include the tumor protein 53 (p53)–nuclear factor erythroid 2–related factor 2 (Nrf2) signaling network, the GTP cyclohydrolase 1/tetrahydrobiopterin (GCH1/BH_4_) pathway, the dihydroorotate dehydrogenase (DHODH)/CoQ_10_ pathway, and the ferroptosis suppressor protein 1 (FSP1)/CoQ_10_ pathway.

#### 2.4.1. p53-Nrf2 Signaling Network

p53 is a tumor suppressor gene triggered by cellular stress, and mediates cell growth arrest [56]. Its function in ferroptosis is distinct from the effects mentioned above. It enhances ferroptosis sensitivity by blocking the uptake of cystine and decreasing intracellular GSH synthesis mainly through suppressing the system Xc^-^ critical subunit SLC7A11 expression [57]. Nrf2 is a core transcription factor in the response to oxidative stress. It undergoes ubiquitination degradation mediated by KEAP1 to maintain low levels under normal physiological conditions. However, structural alterations in KEAP1 during stress conditions lead to elevated levels of Nrf2, substantial nuclear translocation, and activation of downstream antioxidant gene transcription [58]. The downstream activation of Nrf2 involves three major classes of genes for iron metabolism, intermediary metabolism, and GSH synthesis, including FTL, FTH1, HO-1, GPX4, and SLC7A11 [59,60,61]. Normally, Nrf2 activation inhibits ferroptosis by enhancing antioxidant capacity, and conversely, its inactivation promotes the ferroptosis process [62,63,64]. Notably, there are complex interactions between p53 and Nrf2 [65,66]. On the one hand, p53 can directly or indirectly influence Nrf2 activity. For example, fat mass and proteins linked to obesity can demethylate the p21/Nrf2 axis, either p53-dependently or non-dependently [67]. Additionally, the activation of Nrf2 can antagonize the pro-ferroptosis effects of p53, showing antagonistic or synergistic relationships at different stress intensities [68]. Additionally, Jose E et al. discovered that p53 and Nrf2 also exhibit synergistic regulation at the cellular cycle level. Both agents repair damage by activating antioxidant defenses and arresting the cell cycle at low concentrations of H_2_O_2_; however, under high levels of oxidative stress, this synergistic effect is suppressed, and cells instead initiate the apoptotic pathway [69]. In summary, ferroptosis is induced by p53 through the downregulation of the expression of SLC7A11, and it is inhibited by Nrf2 through the activation of downstream antioxidant genes. These functions are dependent on the cell type and stress state.

#### 2.4.2. FSP1/CoQ_10_ Pathway

FSP1/CoQ_10_ pathway regulates cytoplasmic redox state independently of the system Xc^-^/GSH/GPX4 pathway [70]. FSP1, a crucial ferroptosis inhibitor, reduces CoQ_10_ to reduced coenzyme Q_10_ (Ubiquinol, CoQ_10_H_2_) utilizing extra-mitochondrial CoQ_10_ or exogenous vitamin K and NAD(P)H/H^+^ as an electron donor, which then scavenges the LOP and inhibits ferroptosis [71]. This pathway has demonstrated a synergistic effect with GPX4 and GSH in maintaining cell membrane redox homeostasis.

#### 2.4.3. DHODH/CoQ_10_ Pathway

The DHODH/CoQ_10_ pathway mainly regulates mitochondrial redox homeostasis to maintain normal mitochondrial physiological functions (mitochondrial ROS production, altered membrane potential, mitochondrial fusion, fission, and autophagy all affect the process of ferroptosis), and it directly affects the sensitivity of cellular ferroptosis [72,73]. Anchored at the inner mitochondrial membrane, DHODH oxidizes dihydroorotate to orotate while reducing CoQ_10_ to CoQ_10_H_2_, a reaction that keeps the mitochondrial CoQ_10_ pool balanced, restrains L-ROS production, and sustains mitochondrial function [74]. DHODH deficiency or decreased activity impairs mitochondrial CoQ_10_ reduction, induces LPO, and leads to ferroptosis. Furthermore, in a GPX4-deficient model, DHODH inhibitors significantly enhanced ferroptosis sensitivity, confirming that this pathway acts independently of GSH metabolism [74,75].

#### 2.4.4. GCH1/BH_4_ Pathway

Similar to the DHODH/CoQ_10_ pathway, the GCH1/BH_4_ pathway is independent of the system Xc^-^/GSH/GPX4 pathway, and both synergistically regulate the mitochondrial redox state. BH_4_ is a multifunctional endogenous cofactor that functions as a potent antioxidant, particularly in protecting against mitochondrial damage, by directly scavenging free radicals, preventing oxidative damage, and maintaining the integrity of mitochondrial membranes [73]. GCH1 acts as the first rate-limiting enzyme in the synthesis of BH_4_, and its activated form protects against ferroptosis [76]. Up-regulation of GCH1 expression promotes antioxidant BH_4_ production and inhibits ferroptosis [77,78]. This pathway often synergizes with the DHODH/CoQ_10_ pathway to regulate mitochondrial redox homeostasis and prevent ferroptosis in cells.

Overall, ferroptosis is governed by a complex, multi-layered regulatory network. The system Xc^-^/GSH/GPX4 pathway is the primary defense pathway, while the p53-Nrf2 signaling network and pathways like FSP1/CoQ_10_ pathway, DHODH/CoQ_10_ pathway, and GCH1/BH_4_ pathway play complementary and synergistic roles in various cellular environments to jointly construct a cellular antioxidant defense barrier in various cellular environments.

## 3. Myocardial Fibrosis

### 3.1. Etiology and Mechanism of Myocardial Fibrosis

The abnormal buildup of collagen and other proteins in the heart’s extracellular matrix is the core pathological feature of MF, which leads to decreased myocardial compliance and cardiac dysfunction, ultimately inducing cardiac remodeling [79]. CFs are crucial in this process, as they synthesize and deposit ECM components in large quantities in response to pathological stimuli [80]. Continuous activation of fibroblasts not only destroys myocardial elasticity but also creates a vicious cycle through the dual feedback of mechanical and chemical signals [81]. The molecular mechanisms can be summarized in three interrelated regulatory mechanisms: ① Dysregulation of the mechanical stress-sensing system, as evidenced by persistent activation of the integrin/focal adhesion kinase (FAK) signaling pathway [82]. Integrin complex formation with FAK is a major mechanosensing organelle in fibroblasts [83]. This process further exacerbates fibrosis by upregulating the transforming growth factor-beta/Smad and Mad-related proteins (TGF-beta/Smad and Smads) pathway [84]. ② Dysregulated energy metabolism, epigenetic reprogramming, TGF-β signaling, and DNA damage can form a vicious, self-reinforcing cycle [85]. Manifestation of sustained TGF-β signaling, inhibition of related repair gene expression in cardiomyocytes, and concomitant activation of NADPH oxidase 4 [86,87]. ③ A chronic inflammatory loop is created when the immune microenvironment becomes disrupted, and the ratio of pro-inflammatory cytokines to anti-inflammatory mediators is unbalanced [88,89]. Significantly elevated pro-inflammatory cytokine levels are linked with adverse clinical outcomes [90]. These multilevel pathologic changes ultimately lead to irreversible damage to cardiac structure and activity.

### 3.2. The Mechanism of Ferroptosis in Myocardial Fibrosis

**Figure 2:**
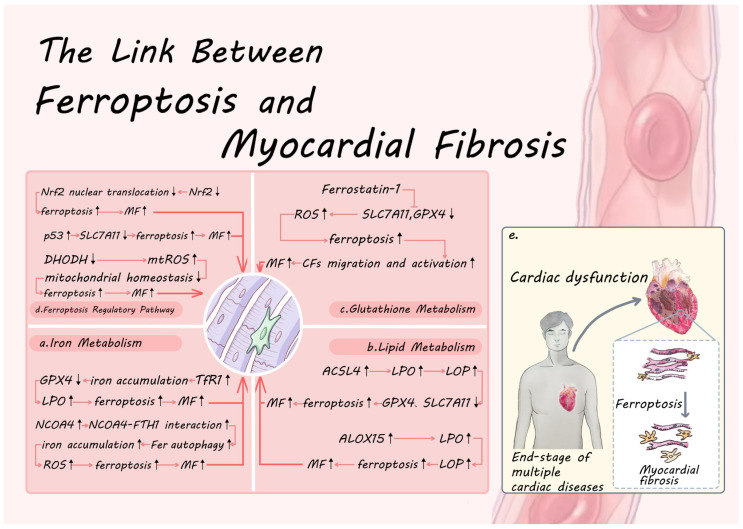
Ferroptosis Mechanism In Myocardial Fibrosis. (**a**–**d**): Connections between ferroptosis and myocardial fibrosis in iron metabolism, lipid metabolism, glutathione metabolism, and ferroptosis regulatory pathways. (**e**): Schematic diagram of tissue-level ferroptosis involvement in the myocardial fibrosis process. Abbreviation: GPX4: Glutathione peroxidase 4; TfR1:Transferrin Receptor 1; LPO: Lipid peroxidation; MF: Myocardial fibrosis; NCOA4: Nuclear receptor coactivator 4; FTH1: Ferritin heavy chain 1; Fer: Ferritin; ROS: Oxygen species; ACSL4: Coa synthetase long-chain family member 4; SLC7A11: Solute carrier family 7 member 11; ALOX15: 15-Lipoxygenase; CFs: Cardiac fibroblasts; Nrf2: Nuclear factor erythroid 2–related factor 2; p53: Tumor protein 53; DHODH: Dihydroorotate dehydrogenase; mtROS: Mitochondrial reactive oxygen species.

Ferroptosis has been newly determined to be crucial to the development of MF [9]. Its regulatory mechanisms encompass iron metabolism, lipid metabolism, GSH metabolism, and multiple ferroptosis regulatory pathways that form a complex network of interactions with MF (Figure 2).

#### 3.2.1. The Link Between Iron Metabolism and Myocardial Fibrosis

Several investigations have demonstrated that [91] iron metabolism can influence MF by inducing ferroptosis. In vitro and animal model studies further revealed that targeting critical features of iron metabolism (like HO-1, Tf) modulates ferroptosis with corresponding changes in the degree of MF [92,93]. Based on the above theories, the fundamental components of iron uptake and iron storage/release, which are two critical facets of iron metabolism, may play a significant role in directing interventions aimed at iron metabolism in MF.

TfR1, a key protein in iron uptake, can regulate ferroptosis by interfering with its molecular number and expression profile [94,95]. It has been demonstrated that *miR214-3p* plays a role in suppressing the expression of TfR1 and FTH1, leading to a decrease in Fe^2+^ deposition. Additionally, it enhances GPX4 expression while inhibiting ACSL4 and COX2 expression, as well as reducing LPO and ferroptosis. These effects contribute to the enhanced cardiac function and myocardial fibrosis in diabetic cardiomyopathic mice [96]. Forkhead box O3 binds to the TfR promoter, represses gene transcription, reduces LOP, inhibits ferroptosis, and ameliorates MF by inhibiting the TGF-β1/Smad2 pathway [97]. In addition, some teams have utilized bioengineering techniques to achieve targeted therapeutic effects with the help of TfR1 protein nanocarriers [98,99]. Based on the extracellular accessibility and internalization ability of TfR1, it has become an ideal target for antibody-mediated anticancer therapy, and it is expected to achieve targeted therapy in the future MF [100].

Iron metabolism and ferroptosis are significantly influenced by NCOA4, a chief regulator that mediates ferritinophagy. Current research on NCOA4 is centered on two facets. One facet is the regulation of ferroptosis through multiple post-translational modifications (PTMs) that modulate NCOA4 bioactivity and affect NCOA4 expression. Ataxia telangiectasia mutated (ATM) kinase promotes ferroptosis by phosphorylating NCOA4 and facilitating NCOA4-Fer interactions, thereby maintaining ferritinophagy [101]. The human YTH domain family 2 selectively binds m6A-modified NCOA4 mRNA and accelerates its degradation, thereby inhibiting ferritinophagy and suppressing cardiomyocyte ferroptosis [102]. Secondly, the intensity of the interaction between NCOA4 and FTH1 directly influences the rate of ferritinophagy and the amount of free iron. Targeted intervention in this interaction can modulate the process of ferroptosis. Disruption of the NCOA4-FTH1 interaction reduces intracellular free iron levels and thus blocks ferroptosis [103]. Based on this property, Zhang Y’s team developed novel nanoparticles targeting NCOA4 to enhance NCOA4-FTH1 interaction and induce ferroptosis [104]. The mechanistic studies mentioned above indicate that targeting NCOA4 may serve as a viable strategy for intervening in MF. Research has demonstrated that the suppression of prorenin receptor expression, along with inhibiting NCOA4 expression, results in the inhibition of ferroptosis and enhancement of cardiac function in diabetic cardiomyopathy and MF [105]. Silencing of the fibronectin gene inhibits NCOA4 expression and improves MF [106]. PM2.5 exposure, on the other hand, upregulated NCOA4 expression levels and promoted ferritinophagy and Fe^2+^ release, leading to ferroptosis and MF [12]. Consequently, targeting NCOA4 could be a potential translational medicine target to address iron overload-related MF, given its dual regulatory properties at the level of PTMs and protein–protein interactions.

The above evidence presents two complementary ferroptosis regulatory axes, iron uptake-TfR1 and iron storage/release-NCOA4, which determine the ferroptosis threshold in cardiomyocytes from upstream iron supplementation and downstream iron release, respectively. Iron uptake-TfR1 and iron storage/release-NCOA4 are two complementary axes of ferroptosis regulation, which determine the ferroptosis threshold of cardiomyocytes from upstream iron supplementation and downstream iron release, respectively. Theoretically, blocking both TfR1-mediated iron import and NCOA4-mediated Fer degradation would attack the cellular iron supply from two major pathways, potentially creating a synergistic anti-ferroptosis effect.

#### 3.2.2. The Link Between Lipid Metabolism and Myocardial Fibrosis

Lipid metabolism is involved in the MF process by regulating ferroptosis. Based on the key driving role of LPO in ferroptosis, modulating lipid metabolism is a promising strategy to intervene in ferroptosis and could be an effective approach for MF therapy [107]. Targeting lipid metabolism to regulate ferroptosis can be broadly divided into two main aspects, which include regulating the synthesis of PL-PUFA and blocking the chain reaction of LPO.

Disrupting the metabolism of PUFAs and their incorporation into phospholipids (PL-PUFAs) can significantly alter a cell’s sensitivity to ferroptosis. This provides opportunities to modulate ferroptosis at the substrate level [108]. ACSL4, an enzyme crucial to the PUFA biosynthesis, participates substantially in ferroptosis. Inhibition of ACSL4 expression specifically inhibits AA esterification, thereby blocking the ferroptosis process [40]. Intervention of ACSL4 expression by either PTMs or gene expression affects lipid metabolic processes [37,109]. It was shown that Coactivator-associated arginine methyltransferase 1 maintains ACSL4 protein homeostasis and inhibits ferroptosis by directly methylating ACSL4 (R339 site) and driving the subsequent process of ubiquitination of ACSL4 [110]. By directly phosphorylating ACSL4 (S447 site) and promoting ACSL4 degradation, cyclin-dependent kinase 1 lowers PUFA synthesis and prevents ferroptosis [111]. Upon sensing LPO signaling, protein Kinase C beta II (PKCβII) is activated and phosphorylates ACSL4, triggering a positive-feedback loop that accelerates PUFA-containing lipid synthesis and expands LPO pools. This process is a key driver of ferroptosis [112]. Increased expression of mitochondrial aldehyde dehydrogenase inhibited the expression of ACSL4 and NCOA4, while increasing expression of GPX4 and SLC7A11, thus inhibiting ferroptosis and enhancing cardiac function in a mouse model of Alzheimer’s disease with MF [113]. It was shown that Mitophagy receptor FUNDC1 knockdown increased ACSL4 expression and promoted LOP accumulation, while decreasing GPX4 and SLC7A11 expression and exacerbating cardiac dysfunction in septic mice with MF [114]. In conclusion, down-regulation of ACSL4 by gene expression intervention or PTM intervention impaired PL-PUFA production at the substrate level, thereby significantly reducing cardiomyocyte sensitivity to ferroptosis.

Targeted inhibition of specific enzymes involved in the initiation of LPO can block the cascade leading to ferroptosis, without disrupting normal lipid metabolism. The strategy focuses on key enzymes in the ferroptosis pathway [115]. A crucial component of the LOX family, ALOX15, significantly participates in the [43]. Fibrosis [116], cancer [117], cardiovascular disease [118], and neurodegenerative diseases [119] are among the diseases linked to ferroptosis that exhibit elevated ALOX15 expression. Inhibiting ALOX15 expression or silencing its upstream genes can alleviate the symptoms of ferroptosis. Several investigations have used bioengineering techniques to target ALOX15 to promote ferroptosis for cancer therapy [120,121]. Research demonstrates that transgenic mice that overexpress the ALOX15 gene experience increased inflammation and ferroptosis, which leads to MF, cardiac dysfunction, and heart failure [122]. As evidenced in in vitro and in vivo experiments, delphinidin improves cardiac function in MF by inhibiting cardiomyocyte apoptosis and ferroptosis. It achieves this by using molecular docking to bind with and degrade the ferroptosis-related protein ALOX15 [123]. Therefore, modulating ACSL4 and ALOX15 is crucial to address lipid metabolism-associated MF. Therefore, precise inhibition of ALOX15 at the LPO onset stage can effectively curb the cascade amplification of LPO without affecting the homeostasis of systemic lipid metabolism, and provide a druggable intervention for MF treatment.

In summary, the two strategies of substrate restriction and chain blockade, targeting ACSL4 and ALOX15, respectively, can create a complementary intervention network in a multidimensional direction and achieve the synergistic inhibition of iron-death-associated MF.

#### 3.2.3. The Link Between Glutathione Metabolism and Myocardial Fibrosis

GPX4 and the system Xc^-^ core protein SLC7A11, as classical regulators of ferroptosis, have been identified as key targets of ferroptosis. Targeting these proteins can manipulate the ferroptotic cell death pathway, offering a potential therapeutic strategy for MF [124,125]. While SLC7A11 gene knockdown exacerbates MF and cardiac dysfunction, *tsRNA-5008a* gene knockdown targets and elevates SLC7A11 expression, significantly suppresses ferroptosis, and alleviates MF both in vivo and ex vivo [126,127]. It has been shown that LRRc17 downregulates GPX4 and SLC7A11 expression, thereby facilitating ferroptosis. It also enhances the migration and viability of CFs, which in turn accelerates the fibrotic process following myocardial infarction and influences ventricular remodeling [11]. Intervention by the glutathione synthetase inhibitor Buthionine sulfoximine disrupts the GSH metabolic cycle, leading to oxidative stress and ferroptosis, which can result in cardiac dysfunction and MF [128]. Ferrostatin-1, a Ferroptosis Inhibitor, attenuates myocardial hypertrophy and MF in mice with right ventricular dysfunction by inhibiting HMOX1/GSH signaling and inhibiting LPO and Ferroptosis [129]. In addition, dexmedetomidine [130], elabela [8], and resveratrol [131] treatments all enhance GPX4 and SLC7A11 expression, block ferroptosis, and thereby lessen MF.

Taken together, GPX4 and SLC7A11 not only share a role in regulating ferroptosis but are also central hubs for redox homeostasis, GSH metabolism, and MF. A pathological cascade involving a disruption in GSH metabolism is followed by amplification of the LPO positive feedback loop, which drives CFs activation and accelerates collagen deposition and ventricular remodeling. Therefore, pharmacological or genetic enhancement of GPX4 and SLC7A11 has the potential to interrupt the signaling of ferroptosis, redirecting it towards fibrotic signaling at the translational level. This action effectively halts the cascade of collagen deposition driven by iron-induced cell death, thereby offering a verifiable molecular target and therapeutic approach for precise intervention in MF.

#### 3.2.4. The Link Between Ferroptosis Regulatory Pathways and Myocardial Fibrosis

The system Xc^-^/GSH/GPX4 pathway and the ferroptosis regulatory pathway collaborate to sustain intracellular antioxidant levels. Its role in MF is mainly reflected in influencing cellular ferroptosis sensitivity by regulating the activity of key molecules (like p53, Nrf2, DHODH), thus providing a potential target for MF intervention.

PTMs and regulatory protein stability are two examples of the complex network of fine-tuning mechanisms regulating p53 activity [57]. Sirtuin 1 and signal transducer and activator of transcription 6 both curb ferroptosis, boost SLC7A11 levels, and block p53 acetylation [132,133]. Gankyrin increases the expression of SLC7A11 and prevents ferroptosis by promoting p53 ubiquitination and degradation [134]. Mitsugumin-53 increases p53 ubiquitination and degradation and raises SLC7A11 and GPX4 levels to reduce cardiomyocyte ferroptosis and improve cardiac function and MF [135]. In regulating p53 protein stability, Musashi Homolog 3A depletion increases p53 protein stability and promotes ferroptosis without altering p53 mRNA levels [136]. Studies have shown that proteasome-activating peptide 1 activates the β5i/p53/SLC7A11 pathway, increases β5i expression, and promotes its binding to p53 and p53 degradation, thereby upregulating the levels of SLC7A11 and GPX4, attenuating oxidative stress, inhibiting ferroptosis, and improving cardiac function and MF [137]. Conclusively, the PTMs and stability regulation of p53 have constituted a highly integrated signaling network, providing critical control points in molecular pathways for precise intervention in MF.

Stability, nuclear translocation, and epigenetic modifications of Nrf2, an antioxidant transcription factor, are critical for regulating ferroptosis. Targeting Nrf2 regulates ferroptosis mainly through PTMs, epigenetic alterations, and modulation of its nuclear translocation level, with PTMs playing a crucial role in controlling Nrf2’s stability, activity, and subcellular localization, thereby finely tuning its antioxidant response and impacting ferroptosis regulation [138]. Obacunone reduces Fe^2+^ and 4-HNE levels, decreases inflammatory cytokine secretion, upregulates GPX4 and SLC7A11 levels, and attenuates ferroptosis injury by inhibiting the ubiquitinated proteasomal degradation of Nrf2 and activating Nrf2 and upregulating its expression [139]. According to findings, DDRGK domain-containing protein 1 hinders the ubiquitinated proteasome from degrading Nrf2, thereby inhibiting ferroptosis in cancer cells [140]. In addition, epigenetic modifications of Nrf2 (like DNA methylation [141], histone modifications [142], and noncoding RNAs [143]) regulate ferroptosis by modulating Nrf2 transcriptional expression through modulation of chromatin structure and gene accessibility. Numerous investigations have demonstrated that Nrf2 functions as a transcription factor, and raising its nuclear translocation level directly elevates the expression of downstream genes (such as GPX4 and SLC7A11) and inhibits ferroptosis [144,145]. Histochrome administered intravenously markedly improved cardiac performance and mitigated MF by activating Nrf2 and its downstream targets, attenuating cytosolic and mitochondrial ROS, preserving GSH pools, and elevating GPX4 activity [146]. Etomidate alleviates MF and inflammation by upregulating Nrf2 levels, promoting Nrf2 nuclear translocation, elevating GSH activity and HO-1 and GPX4 levels, lowering MDA and ACSL4, and reducing the expression of collagen II and α-SMA as well as the secretion of inflammatory factors (IL-6, IL-1β, and TNF-α) [147]. Specific overexpression of caveolin-1 leads to massive binding to Nrf2, inhibition of Nrf2 nuclear translocation, promotion of ROS and LOP accumulation, ferroptosis, induction of myocardial injury in diabetic mice, and MF [148]. In conclusion, Nrf2 participates in the regulation of ferroptosis via various mechanisms, and its protein stability, nuclear translocation dynamics, and epigenetic modifications could be novel targets for intervention in MF therapy.

DHODH is situated within the inner mitochondrial membrane and is involved in the regulation of oxidative stress and LPO in mitochondria. Additionally, it sustains intracellular antioxidant levels in collaboration with system Xc^-^/GSH/GPX4 [149]. Several studies have shown that reduced DHODH expression alters mitochondrial morphology and function, which in turn promotes ferroptosis [150,151]. Protein kinase B activation upregulates DHODH Expression, improves mitochondrial homeostasis, increases GPX4 and SLC7A11 expression, inhibits ferroptosis, and improves cardiac function and MF [152]. Following transverse aortic constriction surgery, estradiol treatment alleviates cardiac hypertrophy and MF in mice by increasing DHODH levels, enhancing mitochondrial function, and suppressing cardiomyocyte ferroptosis [153]. Conclusively, DHODH serves as a central hub in the regulatory network of mitochondrial metabolism, oxidative stress, and ferroptosis, further expanding the theoretical framework and potential strategies for MF-targeted therapy.

Nrf2, p53, and DHODH have demonstrated the value of druggable targeting through pharmacological PTMs, epigenetic regulation, and subcellular re-localization mechanisms that can modulate the antioxidant capacity of cardiomyocytes, thereby interfering with ferroptosis and MF. Chemical or genetic interventions targeting the above facets have shown anti-MF effects in a variety of preclinical models, providing a direct and solid evidence base for subsequent translational studies.

## 4. Discussion

Ferroptosis is an iron-dependent, lipid-peroxidation-driven modality of programmed cell death that is governed by complex processes of intersecting metabolic and signaling circuits. Disruptions in iron handling, LOP accumulation, and GSH balance collectively precipitate ferroptotic cell death. These different systems work together through a network of interactions to control the process of ferroptosis. Correspondingly, the mechanism of ferroptosis in MF is not a simple linear relationship, and its involvement and regulation are beyond the framework of the traditional pathologic mechanism of MF. This paper describes the complex, multidimensional relationship between ferroptosis and MF, suggesting new avenues for research into understanding the pathomechanisms of MF.

Ferroptosis signals myocardial injury to CFs via multidimensional signaling, activating their phenotypic transformation and providing a cytological basis for MF. The complex and nonlinear relationship between ferroptosis and MF is characterized by a positive feedback loop involving several key pathways, notably the TGF-β/Smad and NF-κB signaling cascades. In this loop (modulation of TGF-β levels [154], regulation of macrophage turnover [155], and modulation of NF-κB signaling [156] all regulate ferroptosis processes), ferroptosis and MF promote and amplify each other, fueling disease progression. On the other hand, ferroptosis is accelerated at the cellular and systemic levels by oxidative stress and inflammatory responses to MF. Oxidative stress directly initiates the inflammatory cascade in addition to rapidly amplifying ferroptosis-associated LPO signaling [157]. Oxidative stress, a classical pathway of MF, not only damages cardiomyocytes themselves, but also promotes ECM deposition by activating CFs proliferation and [158,159]. Modulation of oxidative stress levels has been shown to modulate ferroptosis and ultimately influence MF processes [160,161,162]. The release of cellular contents induced by ferroptosis results in the generation of damage-associated molecular patterns (DAMPs), which disrupt the local immune microenvironment and subsequently promote inflammation and MF [163,164]. Several studies have confirmed that intervention in ferroptosis (silencing of TfR1 [97], activation of Sirtuin 1 [165], Empagliflozin treatment [166], or application of ferroptosis inhibitors [167]) significantly improves MF by prohibiting oxidative stress and responses to inflammation. In addition, recent studies have revealed that non-coding RNAs constitute an important regulatory network in the ferroptosis-oxidative stress-inflammation axis: *miR-130b-3p* both promotes GPX4 expression and inhibits ACSL4 activity, thereby reducing the production of L-ROS and suppressing ferroptosis [168]. The overexpression of *miR-214-3p* leads to both the downregulation of p53 and the upregulation of SLC7A11 and GPX4, thereby inhibiting ferroptosis [169]. Unlike traditional single-target interventions, non-coding RNAs exhibit the unique advantage of multi-pathway synergistic regulation, opening up new avenues for the modulation of ferroptosis [170]. In conclusion, focusing on the ferroptosis-oxidative stress-inflammation axis may offer a novel approach to anti-MF treatment, as it is a key component in the development of MF.

Although targeting ferroptosis has shown promising therapeutic potential in preclinical models of MF, translating it into clinically applicable intervention strategies still faces multiple setbacks. The following six areas represent the primary challenges in integrating the available evidence: ① The core pathway of ferroptosis is widely expressed in multiple organs throughout the body, and the lack of cardiac specificity of intervention targets makes systemic intervention highly susceptible to off-target toxicity.② Inhibition of a single ferroptosis pathway, such as the system Xc^-^/GSH/GPX4 axis, can be overcome by compensatory activation of parallel resistance mechanisms like the FSP1/CoQ_10_, GCH1/BH_4_ pathways. This redundancy explains why targeting one component might not be sufficient to induce ferroptosis and highlights the need for multi-targeted therapies to overcome cellular resistance [171]. ③ The lipid peroxidation profiles and iron metabolism characteristics of MF due to different etiologies (ischemia-reperfusion, pressure load, metabolic disorders) vary significantly, limiting the generalization of clinical intervention strategies. ④ The lack of biomarkers that can dynamically reflect the process of iron-death-MF makes it complicated to set doses in early clinical trials, and current efficacy evaluations heavily rely on indicators like histologic collagen ratios and echocardiography. ⑤ Extensive communication exists between CFs and cardiomyocytes and immune cells, but the distribution of existing delivery platforms in the infarct, border, and distal normal zones varies significantly, and cell-type-specific release is not yet possible. ⑥ Existing animal model studies are mostly based on acute or subacute injuries, which differ significantly from the clinically common chronic, multifactorial (comorbid hypertension, diabetes mellitus, obesity) MF, resulting in positive intervention signals that are difficult to reproduce clinically. The ferroptosis-targeted therapy MF is currently at a pivotal stage in its shift from mechanism validation to clinical translation. Future investigations may benefit from: ① Establishing a cardiac-specific, inducible gene-editing animal model to analyze the net effect of different cell types on ferroptosis intervention. ② Development of a real-time assay platform based on iron-dependent redox ratios (like LIP/CoQ_10_H_2_, PE-AA-OOH/PE-AA) or DAMPs to establish a quantitative basis for modeling the dose-exposure-effect relationship. ③ Introducing a multi-omics-artificial intelligence integration strategy to screen for Ferroptosis-MF bioassays that can predict response to therapy, enabling precise stratification of patient subgroups. In conclusion, the therapy targeting ferroptosis for myocardial fibrosis is currently at a pivotal stage, transitioning from validating mechanisms to clinical application. It remains essential to overcome the current obstacles by employing cardiac-specific modeling, dynamic redox markers, and strategies that integrate multi-omics with artificial intelligence to facilitate precise intervention.

To uncover the complex network of interactions between ferroptosis and MF, we methodically sort out the mechanism of ferroptosis in the development of MF from various aspects, such as iron metabolism, LPO, GSH metabolism, and the ferroptosis regulatory pathway. These studies indicate that ferroptosis, through the triad mechanism of iron overload-LPO-antioxidant system imbalance, not only directly exacerbates cardiomyocyte damage but also triggers CF proliferation, facilitates the abnormal deposition of ECM, and disrupts the cardiac immune microenvironment, making it a key pathological factor driving the process of MF. This finding offers an innovative viewpoint on the mechanism of MF and the development of therapeutic targets. In the future, further investigations can be performed to investigate the molecular regulatory network of ferroptosis and its interactions with the classical signaling pathway of MF. We can focus on the modulation at the level of subcellular organelles and intercellular communication, while also striving to enhance the understanding of the pathological mechanisms of MF. By integrating these findings, we aim to establish a solid theoretical framework, ultimately offering a reliable scientific basis for the precise optimization and clinical application of targeted intervention strategies against ferroptosis-related diseases.

## Data Availability

No new data were created or analyzed in this study. Data sharing is not applicable to this article.

## Abbreviations

MF: Myocardial fibrosis; ECM: Extracellular matrix; CFs: Cardiac fibroblasts; PL-PUFA: Phospholipid-based polyunsaturated fatty acids; LOP: Lipid oxidation products; NCOA4: Nuclear receptor coactivator 4; system Xc^-^: Cystine/glutamate antiporter system Xc^-^; GSH: Glutathione; GPX4: Glutathione peroxidase 4; Tf: Transferrin; Fer: Ferritin; LIP: Labile iron pool; FPN: Iron transport protein, Ferroportin; IRE/IRP: Iron-responsive element/Iron-regulatory protein; TfR: Transferrin receptor; TfR1: Transferrin Receptor 1; STEAP2: Six-transmembrane epithelial antigen of prostate 2; STEAP3: Six-transmembrane epithelial antigen of prostate 3; ZIP8/14: Zinc-iron transport protein 8/14, solute carrier family 39 member a8/14; DMT1: Divalent metal transporter 1; HCP1: Heme carrier protein 1; HO-1: Enzyme heme oxygenase-1; FTH1: Ferritin heavy chain 1; FTL: Ferritin light polypeptide; LPO: Lipid peroxidation; L-ROS: Lipid reactive oxygen species; ROS: Oxygen species; PUFA: Polyunsaturated Fatty Acid; AA: Arachidonic acid; AdA: Adrenic acid; PE: Phosphatidylethanolamines; ACSL4: Coa synthetase long-chain family member 4; ALOX15: 15-Lipoxygenase; mtROS: mitochondrial reactive oxygen species; CoA: Coenzyme A; LPCAT3: Lysophosphatidylcholine acyltransferase 3; LOX: Lipoxygenase; COX: Cyclooxygenase; POR: Cytochrome P450 oxidoreductase; SLC7A11: Solute carrier family 7 member 11; SLC3A2: Solute carrier family 3 member 2; GSSG: Glutathione disulfide; GR: Glutathione reductase; p53: Tumor protein 53; Nrf2: Nuclear factor erythroid 2–related factor 2; GCH1/BH_4_: GTP cyclohydrolase 1/tetrahydrobiopterin; DHODH: Dihydroorotate dehydrogenase; FSP1: Ferroptosis suppressor protein 1; CoQ_10_: Coenzyme Q10,Ubiquinone; CoQ_10_H_2_: Reduced Coenzyme Q10, Ubiquinol; FAK: Focal adhesion kinase; TGF-beta/Smad and Smads: Transforming growth factor-beta/Smad and Mad-related proteins; PTMs: Post-translational modifications; ATM: Ataxia telangiectasia mutated; DAMPs: Damage-associated molecular patterns.
